# Antimalarial natural products: a review

**Published:** 2012

**Authors:** Faraz Mojab

**Affiliations:** *Pharmaceutical Sciences Research Center (PSRC) and School of Pharmacy, Shahid Beheshti University of Medical Sciences, Tehran, I. R. Iran*

**Keywords:** Antimalarial plants, Malaria, Natural Products

## Abstract

**Objective: **Malaria is an infectious disease commonplace in tropical countries. For many years, major antimalarial drugs consisted of natural products, but since 1930s these drugs have been largely replaced with a series of synthetic drugs. This article tries to briefly indicate that some plants which previously were used to treat malaria, as a result of deficiencies of synthetic drugs, have revived into useful products once more. It also attempts to describe some tests which can be used to evaluate plant extracts for antimalarial activity.

**Materials and Methods: **By referring to some recent literatures, data were collected about plants used for the treatment of malaria, evaluation of plant extracts for antimalarial activity, modes of action of natural antimalarial agents, and recent research on antimalarial plants in Iran and other countries**.**

**Results and Conclusion: **There is an urgent need for the development of new treatments for malaria. Many countries have a vast precedence in the use of medicinal plants and the required knowledge spans many centuries. Although malaria is controlled in Iran, some researchers tend to study malaria and related subjects. *In vitro* biological tests for the detection of antimalarial activities in plant extracts are currently available. It is vital that the efficacy and safety of traditional medicines be validated and their active constituents be identified in order to establish reliable quality control measures.

## Introduction

Malaria is an infectious disease commonplace in tropical countries with the majority of their population still relying on the use of plant extracts to combat the ravages of the disease. It has been estimated that currently some 1,600 million people live where they can be exposed to malaria and that 400 million people live in countries where malaria is endemic (Phillipson and O’Neill, 1987 ▶). In 1982, it was calculated that approximately 215 million people were chronically infected and that some 150 million new cases were reported each year. The causing agents are 4 species of *Plasmodium*, viz. *P. falciparum*, *P. malariae*, *P. ovale* and *P. vivax*. The latter three species are less dangerous than *P. falciparum* but they persist in the liver and relapses can occur many years after primary infection. However, it is *P. falciparum* that is the cause of malignant tertiary malaria, which is life threatening.

For many years, quinine remained the major antimalarial drug, but from 1930s this natural product was largely replaced by a series of synthetic drugs including 8-aminoquinolines (e.g. primaquine), 4-aminoquinolines (e.g. chlooquine, amodiaquine) and folic acid synthesis inhibitors (e.g. proguanil, pyrimethamine). By the mid 1950s, it was confidently expected that malaria would be eradicated worldwide, but by the mid 1960s this confidence was undermined because of the problems of resistance (Phillipson and O’Neill, 1987 ▶). The vector mosquito developed resistance to potent insecticides such as DDT and certain strains of *P. falciparum* became resistant to chloroquine treatment. By the early 1980s, several strains of *P. falciparum* had become multi-drug resistant and today chloroquine resistance is widespread in S. E. Asia, S. America and E. Africa. With increase in international travel, the problem of malaria does not belong solely to the tropical countries (Phillipson and O’Neill, 1987 ▶).

Chemotherapeutic agents will continue to be in demand for the complete management of malaria and the issue of resistance means that discovering new antimalarial drugs is an urgent priority. In addition to the need for the development of new antimalarial drugs, it is essential to establish the efficacy and safety of traditional medicinal plants which are used to fight the disease. The purpose of this article is to briefly describe some plants that are used to treat malaria, to describe some test systems which can be used to evaluate plant extracts for antimalarial activity, and to consider some of the ongoing related researches.

## Plants used for the treatment of malaria

**Figure1:**
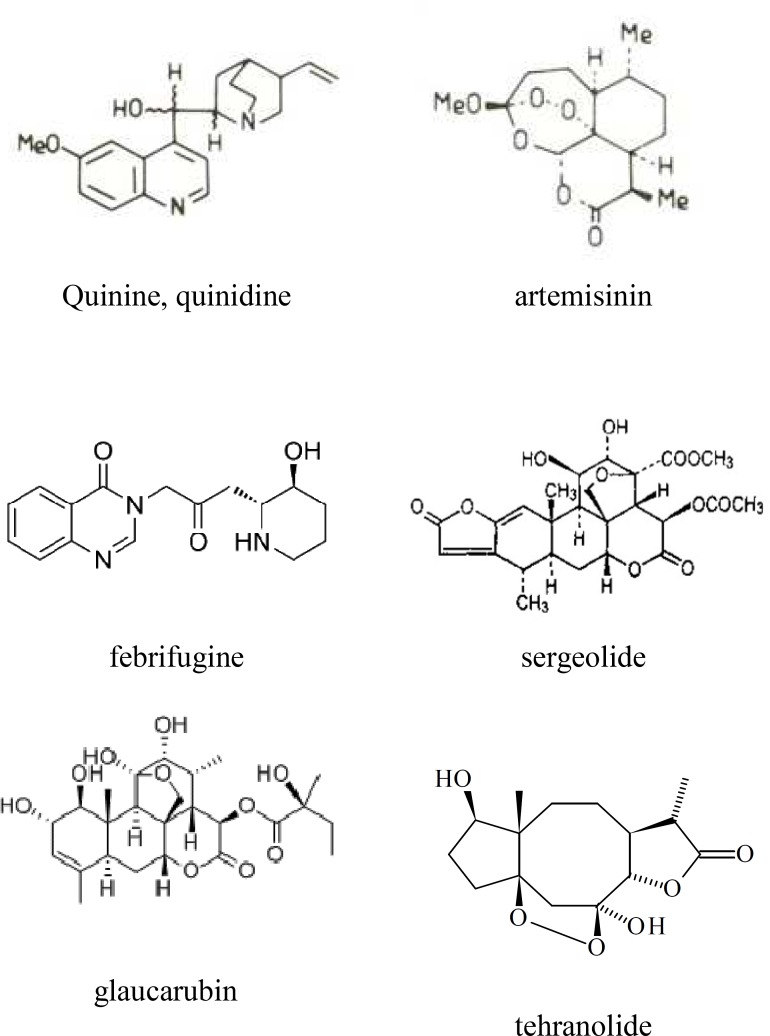
Some examples of antimalarial natural products

*Cinchona* species are well known for their antimalarial properties and the constituent alkaloid quinine is still acknowledged as an effective drug. Perhaps the less widely known stereoisomer quinidine (Figure 1) is at least as potent as, and possibly more potent than quinine (White, 1985 ▶). The Chinese traditional treatment of malaria includes the use of *Artemisia annua* (Compositae) and its active compound, artemisinin, which are currently under considerable interest. (Figure 1) (Phillipson and O’Neill, 1987 ▶). Artemisinin has a higher chemotherapeutic index than chloroquine and is effective in chloroquine-resistant strains of human malaria (Warhurst, 1985 ▶). Another species used as an antimalarial drug in Chinese traditional medicine is *Dichroea febrifuga* (Saxifragaceae) (Anonymous, 1975 ▶). The active principle, febrifugine (Fig. 1) has been used clinically against *P. vivax* and *P. ovale* but its liver toxicity makes it unacceptable as a useful antimalarial drug (Steck, 1972 ▶). The use of plants for the treatment of malaria extends to at least three continents including several countries in Africa (Sofowora, 1980 ▶), Americas (Lewis and Elwin-Lewis, 1971 ▶) and Asia (Chopra et al., 1956 ▶). The NAPRALERT natural product database lists species from 152 genera which have folklore reputations for antimalarial properties. It is important that using modern biological techniques plants with these traditional representations are investigated in order to establish their safety and efficacy, and to determine their value as sources of new antimalarial drugs.

## Evaluation of plant extracts for antimalarial activity

**Figure 2:**
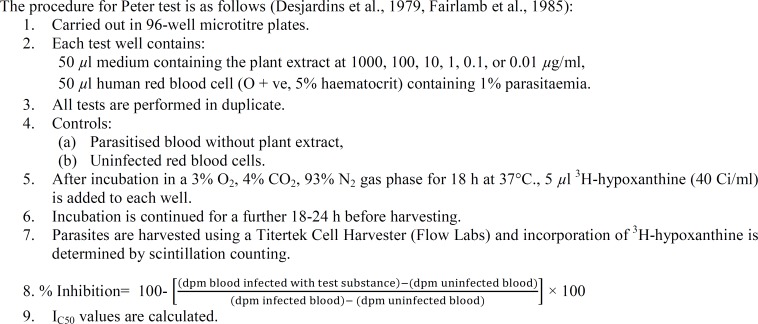
determination of *in vitro* anti-*Plasmodium falciparum* activity in plant extracts.

It is only in recent years that good *in vitro* tests have been established for antimalarial activity. However, previous expensive, time-consuming *in vivo* testing is still required, even for initial screening purposes. The largest published study for the assessment of plant extracts was reported by Phillipson and O’Neill (Phillipson and O’Neill, 1987 ▶). Some 600 plants representing around 126 families were extracted and their extracts tested for their *in vitro* activities against *P. gallinaceum* in chickens and *P.* *chatemerium* and *P. lophurae* in ducklings. Species from over 30 genera yielded extracts which were active against these avian malarias. However, the ability of such tests to predict activity against human malaria is not confirmed yet. Nevertheless, a number of noteworthy observations were made. Particularly, several species of Simaroubaceae which are used in traditional medicine in Africa, Americas and Asia, gives extracts which are active against avian malaria. The most significant advance in antimalarial testing followed the development of a method for the continuous *in vitro* culture of the human malaria parasite, *P. falciparum* (Trager and Jensen, 1976 ▶). In 1979, Desjardins *et al*. described a technique for the quantitative assessment of *in vitro* anti-*P.* *falciparum* activity. The test has since been modified (Fairlamb et al., 1985 ▶), but still relies on the ability to inhibit the incorporation of ^3^H-hypoxanthine into plasmodia. Further details of the *in vitro* test are given in Figure 2.

Guidelines for antimalarial screening of drugs were established by WHO (WHO, 1973 ▶) and four stages were described. Primary screening establishes whether compounds are active against malaria parasites whereas secondary screening sets out to further qualify and quantify antiparasitic activity and to determine safety and comparative activities of analogues. The purpose of tertiary screening is to study non-human and human parasites in primates other than man prior to the fourth stage of the clinical testing. In assessing the activity of plant extracts for the presence of compounds with antimalarial activity, the techniques of primary and secondary screening as outlined by Peters (1987) can be utilized. For initial screening, both *in vivo* and *in vitro* techniques may be employed (Phillipson, 1991 ▶).

Preliminary experiments have been performed in order to determine whether this test would serve to evaluate the activity of crude extracts from plants (O'Neill et al., 1985 ▶). Ethanolic extracts of *Artemisia annua* and of *A. vulgaris* were prepared and the former was shown to contain artemisinin, which was absent from the latter. The IC_50_ values obtained on the basis of tenfold dilution followed by twofold dilutions within narrow ranges of concentration proved to be 3.9 *µ*g/ml and 250 *µ*g/ml, respectively. Hence, the *in vitro* test discriminated between species of the same genus in which the antimalarial compound, artemisinin, was present in only one of the two species.

Crude extracts of three species of Simaroubaceae, namely *Brucea javanica* and *Simaba cedron*, both of which are used in traditional medicine for the treatment of malaria, and of *Ailanthus alitissima*, were prepared for evaluation by the *in vivo* test. Sequential fractionation using solvents of different polarity resulted in petroleum ether, methanol, and aqueous extracts; the methanol extracts were subsequently partitioned between chloroform and water. The three species yielded active extracts and in each case the activity was concentrated in the chloroformic fraction (O'Neill et al., 1985 ▶). The ability of the *in vitro* test to detect active compounds in relatively crude fractions has been further demonstrated by assessing the activity of *B. javanica* fractions obtained from polyamide columns (O'Neill et al., 1985 ▶). Clearly, an *in vitro* test against multi-drug resistant *P. falciparum* that can be used for the evaluation of crude extracts of plants, has considerable value for the assessment of plants used in traditional medicine for the treatment of malaria.

**Figure 3:**
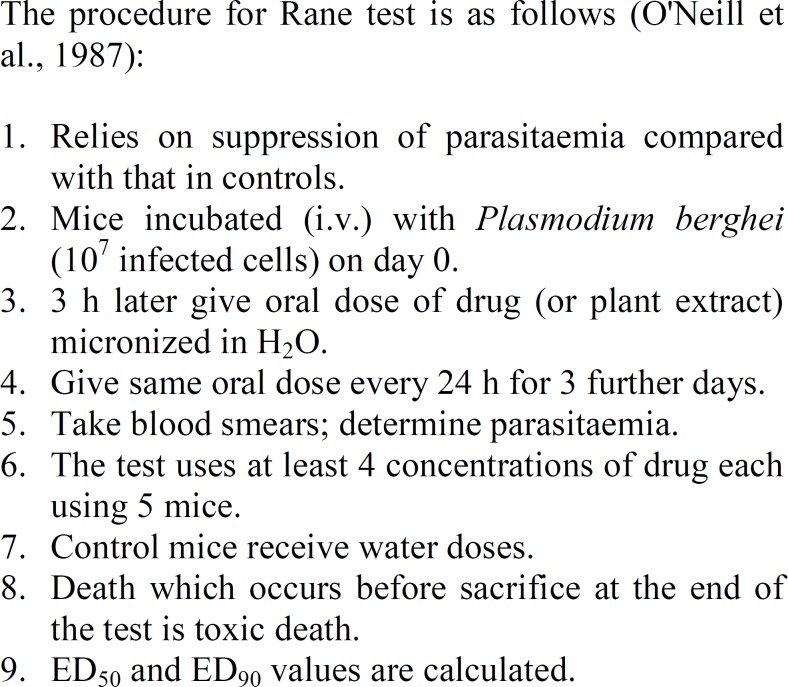
Determination of *in vivo* antimalarial activity against *Plasmodium berghei* infections in mice.

Extracts and isolated constituents selected on the basis of *in vitro* testing should be further studied for their *in vivo* antimalarial activity. Several *in* *vivo* test systems are available but their major drawback is that the used plasmodia are species which do not parasitize humans. *P. falciparum* is a selective human parasite. Nevertheless, useful information regarding *in vivo* antimalarial activity can be obtained by assessing the activity against rodent infections. In particular, the 4-day suppression of parasitaemia test against *P. berghei* infections in mice has been used to evaluate plant extracts (Fandeur et al., 1985 ▶, O'Neill et al., 1987 ▶). An outline of the procedure used in the evaluation is given in Figure 3.

## Mode of action of natural antimalarial agent

The effectiveness of any chemotherapeutic agent is dependent upon a favorable therapeutic ratio, i.e. the drug must kill or inhibit the parasite but have little or no toxicity to the host. Although a large number of natural products have been shown to be able to inhibit the growth of one or more species of protozoa, very few are proved to be selectivity toxic to the parasite. Selectivity depends on differences in biochemistry between the parasite and the host; such a drug can operate on a biochemical target in the parasite that is either absent, or significantly different in the host. However, the mode of action of many natural products with antiprotozoal activities is, at present, unknown and it is possible that some of these may act on biochemical targets unique to protozoa (Wright, 2009 ▶).

## Recent research on antimalarial plants

The most significant recent development in naturally occurring antimalarial drugs is arguably the identification of artemisinin (Figure 1) as the active component of the plant *Artemisia annua*, which is used in traditional medicine as an antimalarial agent (Pillipson and O’Neill, 1987 ▶). This unique sesquiterpene contains an endoperoxide group that appears to be an essential requirement for its activity. It is particularly active *in vivo* against chloroquine-resistant *P. falciparum* and is reported to have relatively low toxicity. However, in the usual dose of 0.6 mg/day for 3 days, the average recurrence rate is more than 10% (Phillipson and O’Neill, 1987 ▶). Due to its highly lipophilic nature, there are inherent problems with its administration as a drug and several derivatives have been prepared, including arthemeter (methyldihydroartemisinin) and sodium artesunate (sodium dihyroartemisinin hemisuccinate). Artemisinin and its two derivatives have been used clinically for the treatment of cerebral malaria in an area where chloroquine resistance was endemic and the cure rate was greater than 90% (Li et al., 1982 ▶). The mode of action is not primarily at the level of nucleic acid synthesis but it appears to inhibit protein synthesis (Gu et al., 1983 ▶).

Like most naturally-occurring therapeutic agents, artemisinin exists in the plant in very small concentration. Chinese workers were unable to find artemisinin in about 30 other *Artemisia* species. In another attempt, a group at the Walter Reed Army Institute of Research studied some 70 species and did not find artemisinin in any of them (Klayman, 1993 ▶).

It is often argued by protagonists of herbal medicine that the total plant extract contains a mixture of substances which act synergistically and hence it is better for a patient to take the whole plant or an extract rather than a single isolated active ingredient. In this context, it is interesting to note that some flavonoids, which do not possess *in vitro* activity against *P. falciparum*, cause a significant reduction in the IC_50_ value of artemisinin when it is assessed for its ability to inhibit the incorporation of ^3^H-hypoxanthine (Elford et al., 1987 ▶). These flavonoids have little effect on the activity of chloroquine under the same test conditions. It is possible that flavonoids, which are present in *A. annua*, may significantly alter the clinical potential of artemisinin in the treatment of chloroquine–resistant malaria. Although endoperoxides appear to be rare as natural products, it is noteworthy that investigation of another antimalarial plant, *Artabotrys hexapetalus* (Annonaceae), has revealed that its active principle, yingzhausu A (Figure 1), also contains an endoperoxide (Xiao, 1983 ▶). Another component containing an endoperoxide is tehranolide and is identified in *Artemisia diffusa* from Iran (Rustaiyan et al., 1989 ▶). 

It is demonstrated that crude extracts of *A. diffusa *inhibits the growth of *P. berghei in vivo *in mice. The microscopic examination of Giemsa stained slides showed a virtual absence of all blood-stage of murine malaria treated with three concentrations of herbal extracts including 27, 2.7 and 0.27 mg/ml. These observations suggest that the active constituents in the extract may be cytotoxic for *P. berghei*, thereby inhibiting their development to the erythrocytic stage. 

The results specifically indicated the inhibitory effects of the *A. diffusa* crude extracts and the fraction which contains sesquiterpene lactones including tehranolide, on the developmental stages of *P. berghei *by decreasing parasitaemia (Rustaiyan et al., 2009 ▶). 

As artemisinin is a complex molecule, much effort has been put into synthesizing compounds based on the 1,2,4-trioxane ring of artemisinin. Many compounds have been produced from artemisinin, some of which have promising *in vivo* activities in animal models (Wright. 2009 ▶). A number of mono- and bicyclic endoperoxides were prepared and tested for antimalarial activity in search of a simplified analogue of the 5-oxygen-substitued 1,2,4-trioxane ring structure of the naturally occurring antimalarial artemisinin (Figure 2). The compounds were assayed in an *in vitro* system for antimalarial activity against chloroquine-susceptible and chloroquine-resistant strains and their antimalarial activity against *P. falciparum* (Rustaiyan et al., 2009 ▶).

Species of Simaroubaceae are used pantropically for the treatment of malaria *in vivo.* The antimalarial activity has been demonstrated for a number of quassinoids, which are bitter upon tasting, biosynthetically degraded triterpenes and are characteristic of the family (Fandeur et al., 1985 ▶, Chan et al, 1986 ▶, Guru et al., 1983 ▶, Pavanand et al., 1986 ▶, Trager and Polonsky, 1981 ▶, O'Neill et al., 1986 ▶). Bruceantin, simalikalactone D, glaucarubinone, soularubinone, and sergeolide have been demonstrated to be markedly active against *P. falciparum in vitro*. Sergeolide reduced virulence in *P. berghei*-infected mice when administered subcutaneously at 0.26 mg/Kg/day but its high toxicity, with a LD_50_ of 1.8 mg/Kg, is indicative of its unsuitability for the curative treatment of malaria (Fandeur et al., 1985 ▶).

A series of 14 quassinoids tested for *in vitro* antimalarial activity were all active, having IC_50_ values below 0.41 *µ*g/ml and 10 of them possessed IC_50_ values less than 0.02 *µ*g/ml (O’Neill et al., 1986). Under the same test condition, chloroquin diphosphate had an IC_50_ value of 0.21*µ*g/ml. The presence and nature of the ester function at C-15 is of importance for *in vitro* antiplasmodial activity. Glaucarubin is about three times more active than chaparrin (Figure 1), whereas glaucarubinone is about twice as potent as halocanthone, and bruceanthin is more than three times more active than brusatol (Figure 1). The A ring substitution pattern is also crucial for activity e.g. glaucarubinone having an *α*, *β*-unsaturated keto function in ring A is over 10 times more active than glaucarubin (Figure 1). Highly active quassinoids may possess either a C-20/C-11 or a C-20/C-13 oxygen bridge (Figure 1). The nature of the C-15 ester function, the A ring substitution, and the oxygen bridge from C-20 to either C-11 or C-13 appear to be crucial for *in vitro* antimalarial activity, and in these respects there are similarities in the structural requirements needed for antileukemic activity. It has observed that for five quassinoids tested, the *in vitro* antimalarial activities paralleled their antileukemic activities. However, in a study with fourteen quassinoids, it was noted that *in vitro* antimalarial activity did not parallel the *in vivo* P-388 lymphocytic leukemia optimal test to control survival values or optimal doses reported previously (Phillipson and O’Neill, 1987 ▶).

The mechanism of action of quassinoids against *Plasmodium* spp. is not understood and warrants further studies. The interesting results reported to date indicate that these compounds require more detailed investigation, particularly in order to exploit differences in specificity between their antiplasmodial and cytotoxic activities.

## Research on antimalarial plants in Iran

Except for identification of tehranolide in *Artemisia diffusa* and its antimalarial effect, (described above (Rustaiyan et al., 1989 ▶)), some other works are (Table 1).

**Table 1:** Research on antimalarial plants in Iran.

**Author**	**Plant**	**Plasmodium**	**Effect**
Hakiminia, 2004	*Cichorium intybus*	*P. berghei*	Doses 0.07 and 0.1 mg/kg were the most effective and provided 0 of parasitaemia in 4^th^ day
Esmaeili et al., 2009	*Glycyrrhiza glabra*	*P. falciparum* and *P. berghei*	antiplasmodial activity
Rustaiyan et al., 2009	*Artemisia diffusa*	*P. berghei *	decreasing parasitaemia, inhibit the growth
Nahrevanian et al., 2010	*Artemisia khorasanica *	*P. berghei*	successfully tested
Ramazaniet al., 2010a	*Artemisia annua *and *A. absinthium*	*P. bergei*	reduced parasitaemia in mice by 94.28% and 83.28%
Ramazani et al., 2010b	*Prosopis juliflora, * *Boerhavia elegans *and *Solanum surattense *	*P. falciparum* chloroquine-resistant and sensitive strains	IC_50_ [less than or equal to] 50 µg/ml
Ramazani et al., 2010b	*Prosopis juliflora, * *Boerhavia elegans *and *Solanum surattense *	*P. berghei*	good antiplasmodial activity

*In vitro* and *in vivo* antiplasmodial tests were carried out on selected plants traditionally used in Iran. Thirty-two plants were extracted with methanol and tested for their *in vitro* (pLDH assay) activity against *Plasmodium falciparum, in vivo *activity against* P. berghei, *and assessed for any cytotoxicity against the human cancer cell line MCF7 and the normal cell MDBK. Extracts from four plants*, Buxus hyrcana, Erodium oxyrrhnchum, Glycyrrhiza glabra, *and* Ferula oopoda *were found to have significant antiplasmodial activity (IC_50_ ranging from 4.7 to 26.6 g/ml). *G. glabra* showed antiplasmodial activity and has selectivity for *P. falciparum* and *P. berghei* when tested on mammalian cells (Esmaeili et al., 2009 ▶).

The purpose of next research was to study five *Artemisia* species from Iran for their *in vitro* and *in vivo* antimalarial property and detection of artemisinin in the active. Dried plants were extracted by 80% ethanol, and total extracts were investigated for antiplasmodial property and artemisinin content by TLC, HPLC, and ^1^H-NMR techniques. Two plants (*A. annua *L. and *Artemisia absinthium *L.) showed good antiplasmodial activity against multidrug resistant and sensitive strain of *P. falciparum*. *A. absinthium *and *A. annua *at concentrations of 200 mg/kg for 4 days reduced parasitaemia in mice infected with *P. bergei* by 94.28% and 83.28%, respectively, but they could not detect artemisinin in all plants studied in this research. The antiplasmodial property of these two herbs is possibly related to essential oils that present in high amounts in their extracts (Ramazani et al., 2010a ▶).

Antiplasmodial activities of extracts of *Boerhavia elegans *and *Solanum surattense *are reported for the first time. The crude ethanolic extracts were tested for *in vitro *antiplasmodial activity against two strains of *Plasmodium falciparum*: K1 (chloroquine-resistant strain) and CY27 (chloroquine-sensitive strain), using the parasite lactate dehydrogenase (pLDH) assay. The antiplasmodial activity of the extracts was also assessed in the 4-day suppressive antimalarial assay in mice inoculated with *P. berghei*. Crude ethanolic extracts showed good antiplasmodial activity and were further fractionated by partitioning in water and dichloromethane. Often plant species assayed, three species: *B. elegans*, *S. surattense* and *Prosopis juliflora *showed promising antiplasmodial activity *in vitro* (IC_50_ [less than or equal to] 50 µg/ml) and *in* *vivo* with no toxicity. The dichloromethane fraction of three extracts revealed stronger antiplasmodial activity than the total extracts (Ramazani et al., 2010b ▶).

The aerial parts of Iranian flora *A. khorasanica *were collected at flowering stage from Khorassan Province, northeastern Iran in 2008. Toxicity of herbal extracts was assessed on mice, and its antimalarial efficacy was investigated on infected *P. berghei* animals. The herbal extract was successfully tested *in vivo *for its antiplasmodial activity through artemisinin composition, which is widely used as a standard malaria treatment. Although, this study confirmed less antimalarial effects of *A.* *khorssanica* against murine malaria *in vivo*, but there are some evidences on improving the infection using this medication (*In vivo *antimalarial effects of Iranian flora *Artemisia khorassanica *against *Plasmodium berghei *and pharmacochemistry of its natural components (Nahrevanian et al., 2010 ▶).

Antimalarial activity of *Cichorium intybus* against *P. berghei* infections in mice (modified Peter’s method) was evaluated, too. Doses 0.07 and 0.1 mg/kg were the most effective and eliminated parasitaemia in the fourth day. Chloroquine was used as a positive control (Hakiminia, 2004 ▶).

## Conclusion

There is an urgent need for the development of novel drugs to treat malaria. Biological investigations into plants used traditionally for primary health care are one obvious way in which searching for new leading compounds should concentrate. Many countries have vast experience in the use of medicinal plants and the required knowledge spans many centuries. *In vitro* biological tests for the detection of antimalarial activity in plant extracts are currently available and some measure of specificity of action can be obtained by monitoring their cytotoxicity in mammalian cells (O'Neill et al., 1986 ▶, Keene et al., 1986 ▶). The biological activities of several compounds isolated from species of the Simaroubaceae have provided interesting leads which require further investigation (O'Neill et al., 1986 ▶, O'Neill et al., 1987 ▶). It is vital that the efficacy and safety of traditional medicines be validated and their active constituents be identified so that reliable quality controls can be established.
